# Supplementary material for: Pituitary-Gland-Based Genes Participates in Intrauterine Growth Restriction in Piglets
Source: Genes (Basel). 2022 Nov 17;13(11):2141. doi: 10.3390/genes13112141 (PMC9690139; doi:10.3390/genes13112141)
Supplement: Supplementary file 1 [file genes-13-02141-s001.zip › Supplementary Figure S1.pdf]

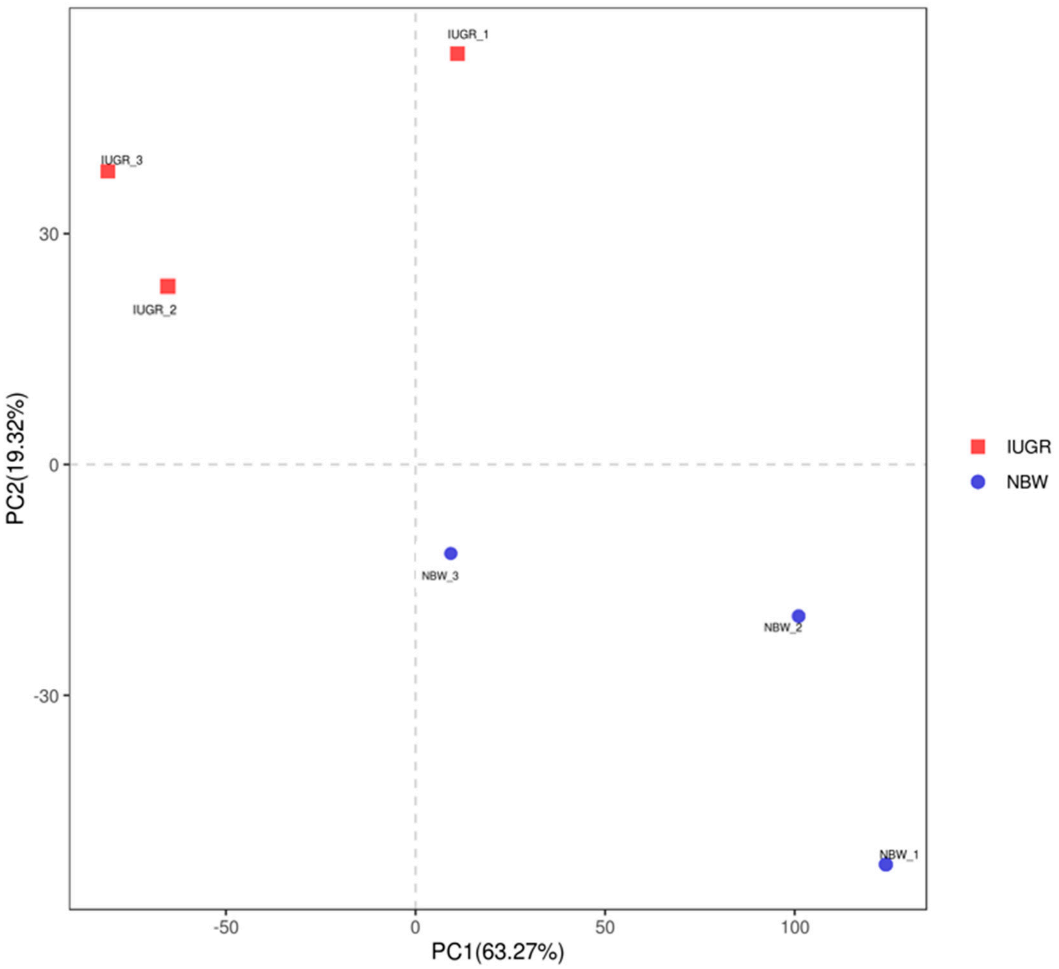

Figure S1. Differences between transcriptome replicates of IUGR and NBW group piglets based on principal component analysis.

Note: the abscissa is the first principal component and the ordinate is the second principal component.
