# Supplementary material for: Pituitary-Gland-Based Genes Participates in Intrauterine Growth Restriction in Piglets
Source: Genes (Basel). 2022 Nov 17;13(11):2141. doi: 10.3390/genes13112141 (PMC9690139; doi:10.3390/genes13112141)
Supplement: Supplementary file 1 [file genes-13-02141-s001.zip › Supplementary Table S1.pdf]

Supplementary Table S1: The results for RNA integrity.

Sample	OD260/280	RIN Value	28S/18S	Result
NBW1	2.012	8.4	1.9	A
NBW2	2.097	8.6	1.8	A
NBW3	2.098	8.0	1.9	A
IUGR1	2.041	8.4	2.1	A
IUGR2	2.054	7.9	1.9	A
IUGR3	2.004	8.5	1.8	A
