# Supplementary material for: Pituitary-Gland-Based Genes Participates in Intrauterine Growth Restriction in Piglets
Source: Genes (Basel). 2022 Nov 17;13(11):2141. doi: 10.3390/genes13112141 (PMC9690139; doi:10.3390/genes13112141)
Supplement: Supplementary file 1 [file genes-13-02141-s001.zip › Supplementary Table S2.pdf]

Supplementary Table S2: Primers for RT-Qpcr

Gene name	Product size(bp)	TM(℃)	Primer sequences (5'to 3')
ACTB	158	60	F:CCTTCTTGGGTATGGAATCCTGT<br>R:CACTGTGTTGGCATAGAGGTCTTAC
IGF2	212	60	F:ATCGTGGAAGAGTGCTGCTT<br>R:CATAGCGGAAGAACTTGCCC
FOSB	162	60	F:GTGAAGTTCAAGTCCTCGGC<br>R:TCACAGAGCAAGAAGGGAGG
EPSTI1	188	60	F:AGAGGCTCTTGGTGACTCAC<br>R:GGTTGACTTTCGCCTTGGAG
UCP2	213	60	F:GCCAACAGACGTGGTAAAGG<br>R:TGGCATTACGAGCGACATTG
ITGA1	154	60	F:TGCCTACAACACCAGAACCA<br>R:GGAGGCTCTTCACTGTCACT
ITGA8	179	60	F:CTTTCACATACCTGCGGCTC<br>R:TCAAAGGGTATCTGCCTGCA
