# Supplementary material for: Pituitary-Gland-Based Genes Participates in Intrauterine Growth Restriction in Piglets
Source: Genes (Basel). 2022 Nov 17;13(11):2141. doi: 10.3390/genes13112141 (PMC9690139; doi:10.3390/genes13112141)
Supplement: Supplementary file 1 [file genes-13-02141-s001.zip › Supplementary Table S3.pdf]

Supplementary Table S3:Information on differential expressed genes

Gene ID	Log2 fold change	P-value	Chromosome	Gene name
ENSSSCG00000031616	2.98	1.22E-09	6	FOSB
Novel00842	-2.34	1.35E-06	6	-
Novel00209	2.14	0.00020	12	-
ENSSSCG00000037572	3.40	0.00023	11	EPSTI1
ENSSSCG00000007398	-2.42	0.00039	17	
ENSSSCG00000011973	1.94	0.00043	13	COL8A1
ENSSSCG00000043543	-	0.00051	6	
ENSSSCG00000014833	-1.54	0.00058	9	UCP2
ENSSSCG00000004464	1.61	0.00059	16	ITGA1
ENSSSCG00000045866	2.47	0.00060	6	
Novel00971	1.96	0.00060	8	-
ENSSSCG00000031307	3.03	0.00060	12	
ENSSSCG00000014799	-1.85	0.00076	9	
ENSSSCG00000005498	1.94	0.00076	1	PAPPA
ENSSSCG00000010727	1.51	0.00094	14	GPR26
ENSSSCG00000002383	1.45	0.00105	7	FOS
ENSSSCG00000024514	2.89	0.00127	16	SHISAL2B
ENSSSCG00000003259	3.24	0.00134	6	OSCAR
ENSSSCG00000050205	1.80	0.00136	14	
ENSSSCG00000021998	1.97	0.00137	5	
ENSSSCG00000029311	2.23	0.00141	14	MYPN
Novel00716	2.12	0.00157	4	-
ENSSSCG00000017749	1.97	0.00171	12	
ENSSSCG00000015268	2.25	0.00181	9	FMO1
ENSSSCG00000036201	2.40	0.00186	16	NPR3
ENSSSCG00000021122	7.26	0.00222	5	TPH2
ENSSSCG00000050684	2.71	0.00224	1	
ENSSSCG00000042873	2.90	0.00228	14	
ENSSSCG00000045796	-3.00	0.00228	9	
ENSSSCG00000005267	1.39	0.00236	1	ANXA1
ENSSSCG00000011911	1.87	0.00251	13	DRD3
ENSSSCG00000007805	-3.32	0.00319	3	ATP2A1
ENSSSCG00000026233	1.43	0.00359	2	
ENSSSCG00000045759	2.29	0.00371	8	
ENSSSCG00000026978	-	0.00392	1	ROS1
ENSSSCG00000011046	1.62	0.00402	10	ITGA8
ENSSSCG00000011905	-3.51	0.00494	13	
ENSSSCG00000013088	2.55	0.00503	2	LRRC10B
ENSSSCG00000001793	2.26	0.00510	7	ADAMTSL3
ENSSSCG00000016848	1.62	0.00533	16	EGFLAM
ENSSSCG00000009004	1.22	0.00540	8	SFRP2

ENSSSCG00000004822	1.59	0.00560	1	ALDH1A3
ENSSSCG00000043028	3.72	0.00565	2	
Novel00915	-1.95	0.00566	7	-
ENSSSCG00000036814	-1.50	0.00576	6	CLEC11A
ENSSSCG00000046072	1.58	0.00587	5	
Novel00098	-	0.00600	10	-
ENSSSCG00000034948	1.26	0.00602	5	RASSF9
ENSSSCG00000048240	1.59	0.00633	1	SAMD5
ENSSSCG00000035715	1.68	0.00636	1	GCH1
ENSSSCG00000016613	1.28	0.00649	18	AASS
ENSSSCG00000001036	-1.95	0.00654	7	TFAP2A
ENSSSCG00000039196	-1.24	0.00661	6	POLR2I
ENSSSCG00000003346	1.75	0.00662	6	
Novel00709	3.55	0.00667	4	-
Novel00210	1.24	0.00713	12	-
ENSSSCG00000012489	1.46	0.00715	X	TRMT2B
ENSSSCG00000013463	-1.28	0.00733	2	LSM7
ENSSSCG00000016898	1.66	0.00734	16	
ENSSSCG00000041354	1.90	0.00737	1	
Novel00302	-1.76	0.00746	14	-
Novel00110	1.29	0.00760	10	-
Novel00047	1.28	0.00761	1	-
Novel00866	1.64	0.00771	6	-
ENSSSCG00000006001	1.28	0.00773	4	ENPP2
ENSSSCG00000048712	4.05	0.00786	15	
ENSSSCG00000012572	1.31	0.00792	X	COL4A5
ENSSSCG00000012571	1.28	0.00809	X	COL4A6
ENSSSCG00000004762	-1.47	0.00809	1	DNAJC17
ENSSSCG00000022236	1.19	0.00821	9	
ENSSSCG00000021873	-1.17	0.00848	7	CRABP1
ENSSSCG00000025535	-2.19	0.00870	7	TFAP2B
ENSSSCG00000008073	1.62	0.00908	3	OMD
Novel00618	-6.58	0.00956	3	-
ENSSSCG00000011721	1.20	0.01018	13	P2RY1
ENSSSCG00000008830	2.00	0.01034	8	CWH43
ENSSSCG00000022230	1.13	0.01042	5	CD9
ENSSSCG00000051042	-	0.01054	3	
ENSSSCG00000050121	1.42	0.01065	14	
ENSSSCG00000001739	2.28	0.01101	7	CRISP1
ENSSSCG00000022679	1.36	0.01128	7	COCH
ENSSSCG00000026748	-1.21	0.01129	3	PLK1
ENSSSCG00000014232	1.34	0.01150	2	LOX
Novel00986	2.09	0.01165	8	-
ENSSSCG00000009220	1.11	0.01182	8	DMP1

ENSSSCG00000021130	-1.26	0.01196	7	RPP21
ENSSSCG00000041987	1.61	0.01198	6	
ENSSSCG00000023374	1.35	0.01199	14	SRGN
ENSSSCG00000031321	1.13	0.01202	5	NR4A1
ENSSSCG00000038110	2.29	0.01204	2	TIFAB
ENSSSCG00000013832	-2.19	0.01239	2	
ENSSSCG00000009152	1.37	0.01242	8	SGMS2
ENSSSCG00000038575	-1.30	0.01253	2	
ENSSSCG00000023915	2.25	0.01258	12	SLC2A4
ENSSSCG00000026211	-1.10	0.01271	7	NRN1
ENSSSCG00000043918	2.55	0.01305	6	
Novel00607	3.91	0.01314	3	-
ENSSSCG00000050545	-3.82	0.01341	14	
ENSSSCG00000003368	2.58	0.01348	6	RNF207
ENSSSCG00000051055	2.45	0.01364	14	
ENSSSCG00000006171	1.24	0.01370	4	CRISPLD1
ENSSSCG00000005379	1.49	0.01377	1	GALNT12
ENSSSCG00000031579	1.09	0.01433	4	PCP4L1
ENSSSCG00000035524	1.12	0.01440	12	WNT9B
ENSSSCG00000012543	3.18	0.01474	X	
ENSSSCG00000001963	1.11	0.01495	7	EGLN3
ENSSSCG00000043814	-1.42	0.01522	1	
ENSSSCG00000010826	1.14	0.01523	10	MTARC2
ENSSSCG00000004885	1.15	0.01526	1	CDH19
ENSSSCG00000010664	1.30	0.01542	14	ENO4
ENSSSCG00000001729	-1.86	0.01557	7	PTCHD4
ENSSSCG00000009735	-1.69	0.01561	14	P2RX2
ENSSSCG00000028203	3.93	0.01564	13	
Novel00246	1.90	0.01570	13	-
ENSSSCG00000048035	4.62	0.01575	6	
ENSSSCG00000013100	1.23	0.01575	2	
ENSSSCG00000022592	1.54	0.01605	2	FIBIN
ENSSSCG00000021885	1.34	0.01610	18	MDFIC
Novel01071	1.70	0.01632	9	-
ENSSSCG00000038188	1.26	0.01640	10	
ENSSSCG00000011125	3.83	0.01656	10	GATA3
Novel01001	2.26	0.01660	8	-
ENSSSCG00000005240	1.09	0.01672	1	DOCK8
ENSSSCG00000032098	-1.19	0.01675	6	
ENSSSCG00000047105	-1.90	0.01680	2	
ENSSSCG00000006308	1.07	0.01685	4	CREG1
ENSSSCG00000036932	2.23	0.01687	15	WNT6
ENSSSCG00000032416	1.63	0.01694	10	RGS1
ENSSSCG00000047527	-	0.01700	2	

Novel00794	-4.71	0.01716	6	-
ENSSSCG00000029227	1.10	0.01727	8	LDB2
Novel00244	1.77	0.01757	13	-
ENSSSCG00000031027	1.77	0.01789	X	IRS4
ENSSSCG00000016516	-1.61	0.01825	18	ATP6V0A4
ENSSSCG00000033432	-1.58	0.01856	7	CHMP4A
ENSSSCG00000008522	1.74	0.01865	3	XDH
ENSSSCG00000015272	-	0.01874	9	OPTC
ENSSSCG00000040617	1.33	0.01874	2	TNFAIP8
ENSSSCG00000043030	4.60	0.01878	6	
ENSSSCG00000004614	-1.04	0.01890	1	UNC13C
ENSSSCG00000001481	1.16	0.01915	7	HCRTR2
Novel00779	-2.66	0.01953	5	-
ENSSSCG00000035392	1.66	0.01970	15	IGFBP2
ENSSSCG00000004789	1.10	0.01995	1	THBS1
ENSSSCG00000005136	3.38	0.02006	1	IFNE
Novel00921	3.38	0.02017	7	-
ENSSSCG00000017520	1.44	0.02084	12	LRRC46
ENSSSCG00000001027	1.11	0.02091	7	BMP6
Novel00096	-3.82	0.02104	10	-
ENSSSCG00000034117	-	0.02116	10	U6
ENSSSCG00000051231	-3.80	0.02135	X	
ENSSSCG00000016997	1.68	0.02136	16	FGF18
ENSSSCG00000006857	1.03	0.02197	4	COL11A1
ENSSSCG00000010691	-1.05	0.02201	14	PLPP4
Novel00748	1.32	0.02206	5	-
ENSSSCG00000009631	3.31	0.02211	14	PEBP4
ENSSSCG00000031712	3.75	0.02216	5	MFAP5
Novel00545	-3.56	0.02216	2	-
ENSSSCG00000040461	1.05	0.02224	2	CDKN1C
ENSSSCG00000017614	1.10	0.02229	12	TRIM25
ENSSSCG00000006877	1.11	0.02236	4	SNX7
ENSSSCG00000017781	1.25	0.02238	12	PIPOX
ENSSSCG00000034811	1.10	0.02296	8	QDPR
ENSSSCG00000032587	-1.13	0.02296	6	
ENSSSCG00000001455	2.02	0.02299	7	
Novel00772	-1.86	0.02303	5	-
ENSSSCG00000028924	-1.13	0.02350	12	AURKB
ENSSSCG00000004632	1.44	0.02361	1	GLDN
ENSSSCG00000036101	-2.57	0.02373	12	
ENSSSCG00000044764	1.17	0.02380	13	
ENSSSCG00000012137	3.05	0.02423	X	BMX
ENSSSCG00000008294	2.46	0.02431	3	ACTG2
Novel00346	1.07	0.02438	15	-

ENSSSCG00000017068	1.49	0.02453	16	FAXDC2
ENSSSCG00000003663	-1.21	0.02500	3	HPCAL1
ENSSSCG00000029296	2.04	0.02512	6	FOXC2
ENSSSCG00000010451	1.29	0.02538	14	IFIT2
ENSSSCG00000016885	1.03	0.02546	16	OXRF4
ENSSSCG00000027266	1.56	0.02572	5	PNPLA3
ENSSSCG00000014117	1.81	0.02592	2	THBS4
ENSSSCG00000017797	4.91	0.02607	12	SLC6A4
ENSSSCG00000035887	1.35	0.02623	4	C8orf34
ENSSSCG00000006206	1.21	0.02625	4	
Novel00411	-3.70	0.02697	16	-
ENSSSCG00000007470	1.20	0.02704	17	RIPOR3
ENSSSCG00000008875	2.02	0.02718	8	RXFP1
ENSSSCG00000049203	-	0.02722	1	
ENSSSCG00000016033	1.02	0.02737	15	GULP1
ENSSSCG00000028139	-1.37	0.02740	3	CCDC154
ENSSSCG00000025345	-1.80	0.02770	12	GUCY2D
Novel00383	1.99	0.02774	15	-
ENSSSCG00000041295	3.28	0.02781	14	
Novel00237	3.29	0.02816	13	-
ENSSSCG00000045386	-3.28	0.02823	8	
ENSSSCG00000002917	-1.06	0.02833	6	NFKBID
ENSSSCG00000000117	1.04	0.02838	5	C22orf23
ENSSSCG00000023351	1.07	0.02855	9	PLA2G4A
ENSSSCG00000028046	1.91	0.02861	10	
ENSSSCG00000028056	1.00	0.02937	6	ZFP36
ENSSSCG00000036820	1.06	0.02943	8	
ENSSSCG00000051283	-	0.02943	8	
ENSSSCG00000013337	1.56	0.03028	2	BBOX1
ENSSSCG00000002400	1.42	0.03033	7	
ENSSSCG00000008557	1.02	0.03045	3	EMILIN1
ENSSSCG00000002829	1.14	0.03053	6	MMP2
ENSSSCG00000010332	2.51	0.03064	14	PLAC9
ENSSSCG00000027476	-1.43	0.03068	17	
ENSSSCG00000010303	1.07	0.03077	14	SYNPO2L
ENSSSCG00000049737	-1.18	0.03109	3	
Novel01031	-2.48	0.03116	9	-
ENSSSCG00000041101	-3.65	0.03117	5	
ENSSSCG00000009002	1.16	0.03119	8	TLR2
ENSSSCG00000014869	1.08	0.03131	9	LRRC32
Novel00539	-2.10	0.03137	2	-
ENSSSCG00000036294	1.09	0.03143	6	NUDT19
ENSSSCG00000038089	1.42	0.03155	13	COL18A1
ENSSSCG00000040631	2.16	0.03161	14	LPL

ENSSSCG00000028996	3.31	0.03166	1	ALDH1A1
ENSSSCG00000016867	2.04	0.03169	16	CCDC152
ENSSSCG00000028157	1.09	0.03193	15	CASP8
ENSSSCG00000039358	1.26	0.03202	13	SUSD5
ENSSSCG00000003403	1.17	0.03209	6	CENPS
ENSSSCG00000012593	1.83	0.03214	X	HTR2C
ENSSSCG00000049435	-3.21	0.03238	14	
ENSSSCG00000051441	1.11	0.03245	13	
ENSSSCG00000033146	1.10	0.03302	5	CD163
ENSSSCG00000006359	1.18	0.03309	4	ADAMTS4
ENSSSCG00000051281	-2.37	0.03320	7	
ENSSSCG00000037669	1.86	0.03327	10	PTPN7
Novel00664	-1.91	0.03356	4	-
ENSSSCG00000017331	-	0.03360	12	
ENSSSCG00000010971	-1.97	0.03380	10	ARID3C
ENSSSCG00000049086	1.44	0.03383	8	
ENSSSCG00000037478	-	0.03391	12	PRR29
ENSSSCG00000042422	-3.26	0.03403	10	
ENSSSCG00000021651	-1.57	0.03411	9	SCN2B
ENSSSCG00000014274	1.23	0.03433	2	PDLIM4
ENSSSCG00000047267	-1.91	0.03436	5	
ENSSSCG00000002277	-1.21	0.03454	7	SPTB
ENSSSCG00000033386	1.52	0.03454	4	
ENSSSCG00000035857	-2.44	0.03457	6	
ENSSSCG00000016200	1.89	0.03486	15	PRKAG3
ENSSSCG00000048093	-	0.03492	4	
Novel00103	3.90	0.03501	10	-
ENSSSCG00000026587	1.36	0.03513	9	BATF3
ENSSSCG00000035293	2.69	0.03516	2	
ENSSSCG00000049464	-1.70	0.03532	2	
Novel01159	1.05	0.03542	X	-
ENSSSCG00000040885	-2.71	0.03544	7	
ENSSSCG00000017981	2.38	0.03555	12	ALOXE3
Novel00968	-1.55	0.03559	8	-
ENSSSCG00000002275	1.09	0.03565	7	PPP1R36
ENSSSCG00000035988	5.85	0.03568	15	FEV
ENSSSCG00000047414	-1.76	0.03586	7	
ENSSSCG00000016609	1.33	0.03601	18	SLC13A1
ENSSSCG00000037634	-	0.03614	7	FOXA1
ENSSSCG00000000275	-1.09	0.03619	5	MAP3K12
ENSSSCG00000001419	-1.36	0.03624	7	SLC44A4
ENSSSCG00000022351	1.26	0.03628	14	GSTO1
ENSSSCG00000049585	-	0.03628	9	
ENSSSCG00000036096	-2.57	0.03645	3	

ENSSSCG00000016872	1.10	0.03695	16	HMGCS1
Novel00560	2.47	0.03725	2	-
ENSSSCG00000039057	-1.80	0.03739	3	ALKAL2
ENSSSCG00000018513	1.81	0.03752	2	
ENSSSCG00000034503	1.20	0.03759	15	ERMN
ENSSSCG00000004001	-1.85	0.03768	6	
ENSSSCG00000011766	1.28	0.03849	13	PEX5L
ENSSSCG00000017037	-	0.03861	16	FABP6
Novel00851	-1.18	0.03862	6	-
ENSSSCG00000034488	-4.28	0.03874	14	NEUROG3
ENSSSCG00000048819	-	0.03880	8	
Novel00810	-1.22	0.03920	6	-
ENSSSCG00000030709	-2.60	0.03964	3	
ENSSSCG00000004012	1.06	0.03995	1	THBS2
Novel00107	-1.87	0.04013	10	-
ENSSSCG00000005584	1.09	0.04021	1	CRB2
ENSSSCG00000012295	-1.04	0.04025	X	MAGIX
ENSSSCG00000021511	-4.58	0.04043	13	GCSAM
ENSSSCG00000024290	1.43	0.04068	4	FSBP
ENSSSCG00000012018	1.02	0.04078	13	CHODL
Novel00275	-3.12	0.04084	13	-
ENSSSCG00000013433	1.46	0.04113	2	ADAMTSL5
ENSSSCG00000011723	1.36	0.04128	13	MME
ENSSSCG00000036748	-1.81	0.04157	3	
ENSSSCG00000003078	4.20	0.04166	6	CEACAM19
ENSSSCG00000014238	2.00	0.04189	2	PRDM6
Novel00470	-2.74	0.04222	18	-
ENSSSCG00000014766	1.25	0.04227	9	OR51E1
ENSSSCG00000045479	2.41	0.04246	8	
ENSSSCG00000031493	1.17	0.04248	6	CYBA
Novel00414	-3.05	0.04287	16	-
ENSSSCG00000001439	2.10	0.04302	7	GPSM3
ENSSSCG00000039024	-1.18	0.04333	3	SPSB3
ENSSSCG00000042589	-1.56	0.04369	4	
ENSSSCG00000022248	-2.83	0.04397	13	OTOL1
ENSSSCG00000008796	1.62	0.04410	8	RBM47
ENSSSCG00000016859	2.53	0.04419	16	C7
Novel00905	-1.24	0.04421	7	-
ENSSSCG00000015333	2.72	0.04444	9	ASB4
ENSSSCG00000046950	1.53	0.04493	7	
ENSSSCG00000040931	1.66	0.04524	17	SLC17A9
ENSSSCG00000012852	-1.70	0.04573	2	CDHR5
ENSSSCG00000033518	1.11	0.04602	12	LLGL2
ENSSSCG00000038945	1.21	0.04606	7	

ENSSSCG00000041615	-2.29	0.04619	6	
ENSSSCG00000003600	1.60	0.04619	6	TINAGL1
ENSSSCG00000017548	-1.28	0.04661	12	NGFR
ENSSSCG00000047396	-2.51	0.04669	5	
Novel01000	2.03	0.04670	8	-
ENSSSCG00000044784	1.46	0.04676	4	
ENSSSCG00000004133	1.15	0.04684	1	FUCA2
ENSSSCG00000041917	1.93	0.04700	11	
ENSSSCG00000011360	-1.17	0.04763	13	CELSR3
Novel00056	-2.83	0.04775	1	-
ENSSSCG00000048886	1.12	0.04817	2	
Novel00571	-1.52	0.04831	3	-
ENSSSCG00000036452	1.69	0.04876	9	LRRC17
Novel00105	1.35	0.04911	10	-
ENSSSCG00000032115	1.11	0.04917	6	OSGIN1
ENSSSCG00000041485	-2.48	0.04922	6	
ENSSSCG00000015896	1.44	0.04938	15	FAP
ENSSSCG00000042529	-	0.04991	3	
